# The roles of sex and gender in child and adolescent mental health

**DOI:** 10.1002/jcv2.12059

**Published:** 2022-01-12

**Authors:** Joanna Martin, Julie A. Hadwin

**Affiliations:** ^1^ Division of Psychological Medicine and Clinical Neurosciences, MRC Centre for Neuropsychiatric Genetics and Genomics Cardiff University Cardiff UK; ^2^ School of Education University of Birmingham Birmingham UK

**Keywords:** gender, mental health, neurodevelopment, sex

## Abstract

Neurodevelopmental and mental health disorders are common in children and young people, and frequently continue to be impairing into adulthood. Biological sex and gender identity are important factors in the likelihood of the development, referral and diagnosis of these disorders, as well as sources of clinical heterogeneity. In this editorial, we highlight that the aetiology of neurodevelopmental and mental health disorders in young people is complex and multi‐faceted. Emerging evidence has implicated sex and gender as playing an important role in understanding the onset and course of psychopathology in childhood and adolescence. Here, we underline the significance of continuing to actively study the roles of sex and gender in the context of outlining potential future research directions and understanding how best to support children and young people.

## CONTEXT AND DEFINITIONS

Neurodevelopmental and mental health disorders often emerge in childhood and adolescence, and can have a persistent impact throughout the lifespan. These disorders are heterogeneous with respect to clinical features, including onset, comorbidity, trajectory and outcomes. Research in developmental psychopathology has recognised the roles of sex and gender in understanding heterogeneity within and across different disorders. *Sex* is generally defined as a binary biological attribute (i.e., female or male) that is assigned at birth, and *gender* as an individual's gender identity. Gender identity reflects a socially constructed expectation that can vary across development, cultures, and communities. It captures a person's inherent sense of being a boy/man or masculine, being a girl/woman or feminine, or a gender experience that may change over time or fall outside of the binary (e.g., agender, gender fluid or non‐binary). This editorial aims to highlight emerging evidence and associated narratives linked to understanding the roles of sex and gender in risk, presentation and symptom course of child and adolescent neurodevelopmental and mental health disorders. In addition, it stresses the importance of the continued development of this evidence base for the improvement of clinical practice in terms of referral, timely diagnosis, and the development of effective prevention and intervention methods for the benefit of children and young people.

## SEX/GENDER AND PREVALENCE

Epidemiological studies consistently report sex differences in the prevalence of neurodevelopmental and mental health disorders in childhood and adolescence. For example, in a large Danish population sample, the cumulative incidence of any disorder in childhood (<6 years) or emerging before early adolescence (<13 years) was higher in males, including neurodevelopmental [e.g., attention deficit hyperactivity disorder (ADHD) and autism spectrum disorder (ASD)], behavioural [oppositional defiant disorder (ODD)/conduct disorder (CD)], and anxiety disorders (Dalsgaard et al., 2020). By late adolescence (<18 years), the overall incidence of disorders was around 15% and similar between males and females, though adolescent females were more likely to be diagnosed with anxiety and mood disorders.

Research examining the role of diversity in gender identity has also found links with poor mental health in children and young people. For example, in a USA population study of 10‐11 year‐olds, parent‐reported behavioural and emotional difficulties and adolescent self‐reported mental health problems (including suicidal thoughts and self‐harm) were more common in individuals who indicated that they felt less like their biological sex (felt gender), expressed an increased desire to be the opposite sex (gender non‐contentedness), or reported not conforming to traditional gender roles (gender non‐conformity; Potter et al., 2021). This study suggested that young people with diverse gender identities may be an especially vulnerable group to monitor in terms of mental health and wellbeing.

## EXPLANATIONS OF SEX/GENDER DIFFERENCES

Recent narratives have considered diagnostic differences in neurodevelopmental and mental health disorders. These suggest that the higher childhood prevalence of neurodevelopmental disorders in males may be partly explained by delays in detection and under‐diagnosis in females. For example, existing diagnostic criteria for ASD may not adequately capture female symptom manifestations, or symptoms may be masked in females (Hull & Mandy, 2017). In the context of ADHD, teachers and parents may have gender‐based biases that lead to decreased referral for ADHD in females (Ohan & Visser, 2009). The lower prevalence of anxiety and mood disorders in adolescent males has been presented through a similar lens, focusing on how biological risk can interact differentially with social and cultural expectations of emotional expression across sex/gender (e.g., Chaplin & Aldao, 2013).

Understanding diagnostic biases is important, although this issue is unlikely to fully explain sex and gender differences in children and young people's mental health. In the following paragraphs we discuss theoretical frameworks in developmental psychopathology to present a complex profile of genetic, environmental, social and cognitive risks that are proposed to interact to increase the likelihood and developmental course of neurodevelopmental disorders and mental health problems. Some studies have highlighted generic risk factors that impact all children and young people, and others have identified risks that are experienced more frequently by either sex/gender leading to different prevalences. Further research has started to demonstrate how different risks are associated with disorders in males and females, leading to sex/gender differences in developmental pathways and outcomes.

### Genetic risk

Sex chromosomes are a clear candidate for study, given that most biological males have one *X* and one *Y* chromosome, whereas most females have two *X* chromosomes. In general, genetic syndromes affecting the sex chromosomes (e.g., Fragile *X* syndrome in males and females, Turner syndrome in females) have widespread effects on emotional, behavioural and cognitive development. However, the aetiology of neurodevelopmental and psychiatric disorders is complex, multi‐faceted, and polygenic, and as such the impact of sex chromosomes needs to be considered in the context of the entire genome.

Twin studies can be used to test for overall qualitative or quantitative sex differences in the contributions of genetic, and shared and non‐shared environmental factors. Childhood‐onset conditions show moderate to high heritability based on family and twin studies, though reported genetic and environmental influences can vary with the child's age, the person reporting symptoms, the measurement used, and sex/gender. For example, heritability estimates in a large Norwegian family study measuring anxiety, as reported by 12–18 year‐olds and their parents, were larger for parent‐reported symptoms and for females. In addition, the influence of non‐shared environmental factors was greater for adolescent self‐reported symptoms (Ask et al., 2014). Further work is needed to confirm whether such sex differences in heritability are robust and what they indicate in terms of the contributions of different genetic and shared or non‐shared environmental sources of risk for anxiety in males and females.

Molecular genetic studies are needed to identify specific causal risk factors that may differ by sex. Few reliable sex differences in child psychiatric phenotypes have been noted to date. One example is that studies have identified a greater likelihood of rare genetic mutations (e.g., protein‐truncating variants or de novo mutations not inherited from parents) in autistic females, which is supported by twin studies that observed a greater familial risk of ASD in siblings of autistic females, compared to siblings of autistic males; this pattern of results has been referred to as the *female protective effect* (review by Hull & Mandy, 2017). According to this hypothesis, females may be less likely to be diagnosed with ASD partly because they are protected (via an as yet unidentified mechanism) and therefore require a greater genetic burden to manifest ASD.

Genetic factors do not operate in isolation. It is also important to consider the impact of sex in the context of *gene‐by‐environment interactions* (e.g., genetic risk buffered by positive environmental circumstances) or the impact of *genetic nurture* (i.e., where parental genetic variation that is not transmitted to a child influences the child's mental health via the family environment). Given that tens of thousands of variants are likely to be involved in risk and variability in neurodevelopment and mental health in children and young people, most sex differences in individual gene and gene‐by‐environment effects are likely to be relatively small and will require very large samples to identify, which are not yet available.

### Environmental, social, and cognitive risk

Research in development and psychopathology has consistently demonstrated a negative impact of perinatal and postnatal adverse childhood experiences (ACEs) on neurobiological, emotional, social, and cognitive development. Links between environmental risks (e.g., economic disadvantage, family adversity, parent stress) with developmental outcomes are typically associated with socialisation processes within the family, which reflect suboptimal parent‐child interactions (e.g., harsh or overprotective parenting). The impact of ACEs has been described as a cascade effect that can lead to multiple adverse experiences, compromising development and increasing the likelihood of mental health problems in children and young people. Studies focusing on the association between environmental adversity and neurodevelopmental and mental health disorders have typically statistically adjusted for sex differences in behaviour, to focus on links between risk factors and outcomes regardless of sex, rather than directly testing for differences. Further research is needed to understand the extent to which environmental risks can be linked to robust sex/gender differences and developmental pathways.

Some research groups have made significant leaps in understanding sex differences in the clinical presentation and developmental pathways associated with specific disorders, providing momentum for the exploration of environmental risk across sex/gender. Findings from the FemNat Consortium, for example, identified sex differences in the diagnostic and developmental profile of a large group of 9–18 year‐olds with a diagnosis of CD, highlighting that girls typically showed a later onset and more severe symptoms, and experienced comorbid internalising disorders in adolescence. In contrast, boys were more likely to be given an earlier diagnosis of CD, alongside increased symptoms of ADHD (e.g., Konrad et al., 2021). While authors have argued for further longitudinal research to identify biological and environmental risks associated with sex differences in symptoms and pathways in CD, they also recognised that findings may underline diverse socialisation experiences in males and females across different contexts. For example, Konrad et al. (2021) raised the possibility that a later and more severe CD symptom profile in females may increase distress in school, leading to the greater likelihood of girls developing comorbid internalising difficulties.

The comorbid adolescent profile in females with a diagnosis of CD sits alongside studies that have pointed to sex differences in cognition and the emergence of internalising disorders in adolescent girls. Developmental research, for example, has identified rumination (i.e., repetitive negative thinking that focuses on causes and symptoms of negative emotions) as a transdiagnostic risk factor that is linked to sex/gender differences in adolescent anxiety and depression. A cross‐sectional study with typically developing Dutch children and adolescents aged 8–18 years identified a temporary disruption in different components of emotional regulation at 12–15 years that included a short‐term increase in self‐reported rumination in males (Cracco et al., 2017). Females, however, self‐reported increased rumination across adolescence, which followed a linear trend. The authors linked this chronic and increasing pattern of rumination in females to a heightened risk for negative affect during this period of development.

Related research focusing on mental health difficulties in young people with diverse gender identities has highlighted risk associated with the communication of identity to family and peers. Negative reported experiences include peer stigmatisation, victimisation, and inadequate family and peer support [e.g., review by Spencer et al. (2021)]. Research has further underlined maladaptive cognitive strategies linked to emotion coping (including rumination) as increasing risk for internalising disorders in this group of young people.

## FUTURE DIRECTIONS

Current research has underlined significant heterogeneity in clinical presentation, comorbidity patterns, neuro‐cognitive difficulties, symptom pathways, and long‐term outcomes of children and young people with neurodevelopmental and mental health conditions. Sex and gender are likely to be important sources of this heterogeneity, and their influences need to be better characterised to improve clinical services and developmental outcomes. Future research should aim to more clearly understand the relative contributions of specific genetic, cognitive and environmental risk factors to children and young people's mental health, and whether these differ depending on sex and gender. Studying the development of gender identity across childhood and adolescence, including the examination of its different aspects and expression is an important future research avenue.

Future research should also aim to improve our understanding of the role that biases play in the referral and diagnostic process associated with symptom differences across sex and gender in samples recruited from the community and child psychiatric services. Studies require an a priori focus on sex and gender to ensure that findings are relevant to young people, regardless of differential prevalence rates. This approach will allow an explicit examination of the roles of sex and gender as potential sources of genetic and clinical heterogeneity. Research would also benefit from increasing sample sizes of child and adolescent clinical populations to complement the growing availability of general population cohorts and adult clinical studies. There is also a need to increase the ancestral diversity of genomic samples to ensure that discoveries are relevant globally and to consider the intersection between ethnicity and sex/gender in clinical studies. Research on sex/gender has the potential to improve clinical practice in terms of timely diagnoses and effective sex/gender‐specific prevention and intervention methods. This focus should be a priority for making research advances in children and young people's mental health.

## CONFLICT OF INTERESTS

Drs Martin & Hadwin are Joint Editors of JCPP *Advances*.

## AUTHOR CONTRIBUTION

**Joanna Martin:** Conceptualization, Writing – original draft, Writing – review & editing. **Julie A Hadwin:** Conceptualization, Writing – original draft, Writing – review & editing

## Data Availability

N/A.
